# Snakebite capsules attenuate *Trimeresurus stejnegeri* venom-induced skeletal muscle injury by inhibiting the ferroptosis pathway

**DOI:** 10.1007/s13205-026-04729-8

**Published:** 2026-03-31

**Authors:** Dan Shao, Jianxin Zheng, Jianzhen Zheng, Meiying Chen, Shaohua He, Shuling Huang, Dan Wen, Hui Wu

**Affiliations:** Emergency Department of the Affiliated Peoples Hospital of Fujian University of Traditional Chinese Medicine, No. 602, Middle 817 Road, Taijiang District, Fuzhou City, 350004 Fujian Province R.P. China

**Keywords:** *Trimeresurus stejnegeri* venom, Snakebite capsules, Ferroptosis

## Abstract

Envenomation by *Trimeresurus stejnegeri* induces severe skeletal muscle injury, and while Snakebite Capsules show therapeutic efficacy, their underlying mechanism remains unclear. This study explores the role of ferroptosis in this pathology and the protective effect of Snakebite Capsules. *T. stejnegeri* envenomation causes significant hemotoxicity and local tissue damage. Despite the clinical efficacy of Snakebite Capsules, their protective mechanism remains incompletely understood, particularly in relation to ferroptosis. Using a rabbit model of *T. stejnegeri* envenomation, we explored the role of ferroptosis and evaluated the therapeutic potential of Snakebite Capsules. Rescue experiments included the use of ferroptosis modulators Ferrostatin-1 and Erastin. Our results demonstrated that the venom activates ferroptosis, as evidenced by skeletal muscle myofibril dissolution, increased reactive oxygen species (ROS), decreased glutathione (GSH) levels, accumulation of malondialdehyde (MDA) and lipid peroxides (LPO), and dysregulation of key ferroptosis-related proteins. Treatment with Snakebite Capsules significantly attenuated skeletal muscle injury, restored redox homeostasis, and normalized the expression of ferroptosis-associated proteins. These findings indicate that ferroptosis activation is a key mechanism in *T. stejnegeri* venom-induced muscle damage. We conclude that Snakebite Capsules confer protection at least partially through modulation of the ferroptosis pathway, identifying ferroptosis as a critical target in treating such envenomations.

## Introduction

Snake venom is a complex mixture of bioactive components secreted by venomous snakes (Vespasiano et al. 2025), including proteases, phospholipases, neurotoxins, and procoagulant toxins, which damage the body through various mechanisms such as tissue necrosis, hemorrhage, neurological dysfunction, and coagulation disorders (Offor and Piater 2024). According to the World Health Organization (WHO), approximately 5.4 million people worldwide suffer from snakebites annually, resulting in around 81,000 deaths and 400,000 cases of permanent disability (Stienstra et al. 2026). The *Trimeresurus stejnegeri*, a common venomous snake in Asia, is notorious for its potent procoagulant activity and tissue-damaging effects (Zhuang et al. 2025). The severe outcomes of* T. stejnegeri* envenomation stem mainly from venom toxins that disrupt normal physiological processes, causing pathological changes including tissue necrosis, vascular damage, and coagulation disorders (Hati et al. 1999; Ojeda et al. 2017). Clinical manifestations include localized swelling, intense pain, extensive bleeding, nausea, vomiting, and dizziness. In severe cases, multiorgan failure and death may occur.

Currently, the primary treatment for *T. stejnegeri* envenomation relies on antivenom serum (Chong et al. 2025), which neutralizes toxic venom components (Sanhajariya et al. 2018). However, antivenom therapy faces several limitations. First, its production and storage require specialized facilities and technical expertise, making it inaccessible in remote or resource-limited regions. Second, antivenom administration may trigger allergic reactions and other adverse effects, and it does not fully counteract the venom’s systemic impact. Snakebite Capsules, a traditional Chinese medicine formulation, have demonstrated promising clinical efficacy in treating *T. stejnegeri* envenomation. Their standardized composition ensures safety and tolerability, along with ease of storage and transportation. Despite their therapeutic potential, the precise mechanisms underlying their effects remain unclear. Therefore, elucidating the pathological mechanisms of *T. stejnegeri* venom and identifying novel therapeutic targets are crucial for developing more effective treatments.

In recent years, ferroptosis, a novel form of regulated cell death (Tang et al. 2021), has been implicated in various diseases (Sun et al. 2023), emerging as a potential therapeutic target (Mou et al. 2019). Ferroptosis is an iron-dependent, lipid peroxidation-driven cell death process characterized by glutathione (GSH) depletion and inactivation of glutathione peroxidase 4 (GPX4), leading to the accumulation of lipid peroxidation products (e.g., MDA and LPO) and cell membrane rupture, hallmark features include mitochondrial alterations (e.g., reduced cristae and increased membrane density), elevated lipid peroxidation, and dysregulated iron metabolism (Jiang et al. 2021). Although the role of ferroptosis in *T. stejnegeri* envenomation remains unexplored, studies on other animal venoms suggest a possible connection (Sun et al. 2024; Zeitler et al. 2021). Certain venom components may disrupt cellular iron homeostasis, causing iron overload and ferroptosis induction. Additionally, iron metabolism regulators, such as transferrin receptor 1 (TFR1) and ferrireductase 1 (STEAP3), may modulate ferroptotic pathways (Abeydeera et al. 2024; Gao et al. 2015).

This study investigated the role of ferroptosis in *T. stejnegeri* venom-induced pathology using the rabbit model bitten by *T. stejnegeri*. We examined ultrastructural changes in skeletal muscle via HE staining and electron microscopy to identify ferroptotic features. Ferroptosis-related markers (ROS, GSH, MDA, LPO, and SOD) were measured to assess lipid peroxidation and antioxidant capacity. Furthermore, qRT-PCR quantified the expression of key ferroptosis regulators to elucidate underlying molecular mechanisms. Additionally, we evaluated the therapeutic effects of Snakebite Capsules on ferroptosis-related indicators, clarifying their mechanism in mitigating venom toxicity. These findings will provide novel insights into *T. stejnegeri* venom pathology, uncovering ferroptosis as a critical mechanism of cellular injury. Moreover, defining the antiferroptotic action of Snakebite Capsules may facilitate the development of combination therapies targeting ferroptosis inhibition.

## Material and methods

### Experimental design

This study was a controlled laboratory animal experiment designed to investigate the role of ferroptosis in *T. stejnegeri* venom-induced skeletal muscle injury and to evaluate the therapeutic potential and mechanism of Snakebite Capsules. The study included multiple experimental groups for comparison, including control, venom-only, venom treated with ferroptosis modulators (Erastin or Ferrostatin-1), and venom treated with Snakebite Capsules. Preparation of *T. stejnegeri* venom and snakebite capsules solutions.

### Preparation of *T. stejnegeri* venom and snakebite capsules solutions

We dissolved 120 mg of lyophilized *T. stejnegeri* venom powder (Huangshan Qishe Technology Co., Ltd.) in physiological saline to prepare a 10 mg/mL solution. Snakebite Capsules are a traditional Chinese medicine formulation approved by the National Medical Products Administration of China (Approval Number: Z20025878). Its main herbal components include Radix Pseudoginseng (30%), Rhizoma Bletillae (20%), Radix Sanguisorbae (15%), Cortex Phellodendri (10%), Rhizoma Coptis Chinensis (10%), Radix Angelicae Sinensis (8%), and Radix Glycyrrhizae (7%). Known active ingredients include notoginsenosides (from Radix Pseudoginseng), bletilla mannan (from Rhizoma Bletillae), and berberine (from Rhizoma Coptis Chinensis), and their anti-inflammatory and antioxidant activities have been confirmed by previous studies (Kumar et al. 2025; Gupta et al. 2024). For Snakebite Capsules preparation, 3 g of capsule contents (Fujian University of Traditional Chinese Medicine Affiliated People’s Hospital) were dissolved in physiological saline to make a 0.3 g/10 mL solution.

### Establishment of rabbit model bitten by *T. stejnegeri*

New Zealand White rabbits (Hangzhou Yuhang Kelian Rabbit Industry Cooperative, License Number: SCXK (Zhejiang) 2022-0008), weighing 2.0–2.5 kg with equal numbers of males and females, were used in this study. The study was conducted from January 10, 2023, to April 15, 2023. Animals were housed under standard conditions: ambient temperature maintained at 16–28 °C, relative humidity 30–70%, and a 12-hour light/dark cycle with illumination intensity between 100 and 200 lx. All rabbits had free access to water and received a quantified diet. Rabbits were randomly divided into groups (three in each group). The skeletal muscle injury model was established by single intramuscular injection of 4 mg/kg venom solution into the left hindlimb quadriceps. The selection of this dose was based on literature reports and pre-experiments: clinical data show that the dose of Trimeresurus stejnegeri venom in a single bite ranges from 1 to 5 mg/kg (Zhuang et al. 2025); pre-experiments indicated that 2 mg/kg induced mild injury, 6 mg/kg caused > 30% mortality, while 4 mg/kg stably induced obvious muscle injury (consistent with clinical manifestations) with < 10% mortality, which is suitable for evaluating the therapeutic effect of drugs. At 24 h post-envenomation, treatment with Snakebite Capsules (0.348 g/kg) was administered via daily oral gavage for 7 consecutive days. Animals were maintained under standard conditions with free access to food and water throughout the experimental period. On day 7, animals were euthanized and left hindlimb skeletal muscle tissues were collected. For each group, tissues from 3 animals were immediately frozen in liquid nitrogen and stored at −80 °C for molecular analyses. Tissues from another 3 animals were processed differently: some fixed in 4% paraformaldehyde for histopathology, others cut into 1 mm³ pieces and fixed in 2.5% glutaraldehyde for electron microscopy.

### ROS staining

10 μm-thick frozen muscle sections were prepared. After delineating the staining area with a hydrophobic pen, 100–200 µL DCFH-DA fluorescent probe working solution (BB-470516, BestBio) was applied and incubated at 37 °C for 40 min in the dark. After removal of staining solution, sections were washed three times with ice-cold PBS (5 min each). Finally, sections were mounted with DAPI-containing solution and imaged under fluorescence microscopy.

### GSH measurement

Blood samples were allowed to clot at room temperature for 2 h or at 4 °C overnight, then centrifuged at 1,000×g for 20 min to obtain serum. GSH levels were determined using a commercial kit (A006-2-1, Nanjing Jiancheng Bioengineering Institute) according to the manufacturer’s protocol. Absorbance at 412 nm was measured using a microplate reader. GSH concentration was calculated as: GSH (µmol/L) = [(sample OD - blank OD)/(standard OD - blank OD)]×standard concentration (20 µmol/L)×dilution factor. Triplicate measurements were performed for each sample.

### MDA measurement

Serum samples were processed as above. MDA content was determined using a commercial kit (A001-1, Nanjing Jiancheng Bioengineering Institute) by measuring absorbance at 532 nm. MDA concentration was calculated as: MDA (nmol/mL) = [(sample OD - blank OD)/(standard OD - blank OD)]×standard concentration (10 nmol/mL) × dilution factor. Triplicate measurements were performed.

### LPO measurement

LPO levels were measured using an ELISA kit (MM-0890R1, Enzyme Immunoassay). Serum samples were processed as described for GSH measurement. In the 96-well plate, 50 µL of standard or sample was added to appropriate wells (blank wells received no sample). After adding 100 µL HRP-conjugated detection antibody to all wells except blanks, plates were incubated at 37 °C for 60 min. Following five washes, 50 µL each of substrate A and B were added for 15-min color development at 37 °C in the dark. The reaction was stopped with 50 µL stop solution, and absorbance at 450 nm was measured within 15 min. A standard curve was generated to calculate sample concentrations.

### HE staining

After preparing graded alcohol solutions (70%, 80%, 90%, 95%) and PBS, paraffin sections were dewaxed in xylene (10 min each in xylene I and II), rehydrated through graded alcohols (5 min each in 100%, 95%, 90%, 80%, 70%), and washed with PBS (3 times). Sections were stained with hematoxylin (7211, Epredia) for 5 min, rinsed with running water, and blued in PBS for 5 min. After 1-min counterstaining with eosin (7111, Epredia) and water rinsing, sections were dehydrated (10 min each in 95% and absolute alcohol), cleared in xylene for 10 min, and mounted with neutral balsam for microscopic examination.

### Masson staining

Fresh working solutions were prepared: Weigert’s iron hematoxylin (equal parts A and B solutions mixed immediately before use) and weak acid working solution (2:1 distilled water: weak acid solution). After dewaxing and rehydration, sections were circled with a hydrophobic pen 5 mm from tissue edges. Sections were stained with Weigert’s solution for 10 min at room temperature in the dark, differentiated in acid alcohol (5–15 s), and rinsed. After bluing in Masson bluing solution (5 min) and distilled water rinse (1 min), sections were stained with ponceau-fuchsin solution (7 min), rinsed in weak acid (1 min), differentiated in phosphomolybdic acid (1 min), and rinsed again. Final staining with aniline blue (50 s) was followed by weak acid rinse, rapid dehydration in 95% alcohol, three changes of absolute alcohol (15 s each), xylene clearing (10 min), and mounting. Collagen fibers appeared blue, muscle fibers red, and nuclei blue-black under microscopy.

### qRT-PCR

Glyceraldehyde-3-phosphate dehydrogenase (GAPDH) was used as the housekeeping gene for normalization. Primers for target genes and GAPDH were designed by Servicebio (Wuhan), added 2× SYBR Premix Ex Taq, Forward Primer (5 pmol/µL), Reverse Primer (5 pmol/µL), cDNA, and dd H2O. The cycling conditions were as follows: 95 °C for 30 s, followed by 40 cycles of 95 °C for 15 s, 60 °C for 30 s.

### Data analysis

The data analysis was performed using GraphPad 7 software (GraphPad Inc., La Jolla, CA, USA), and the results were presented in graphical form. All experiments were conducted in triplicate. Paired data was statistically analyzed using Student’s t-test, and significance levels were denoted as follows: *p < 0.05, **p < 0.01, ***p < 0.001. Error bars in the graphs represent mean ± standard deviation (SD) of three independent biological experiments.

## Results

### Establishment of a rabbit model of *T. stejnegeri* venom-induced skeletal muscle injury

**Fig. 1:**
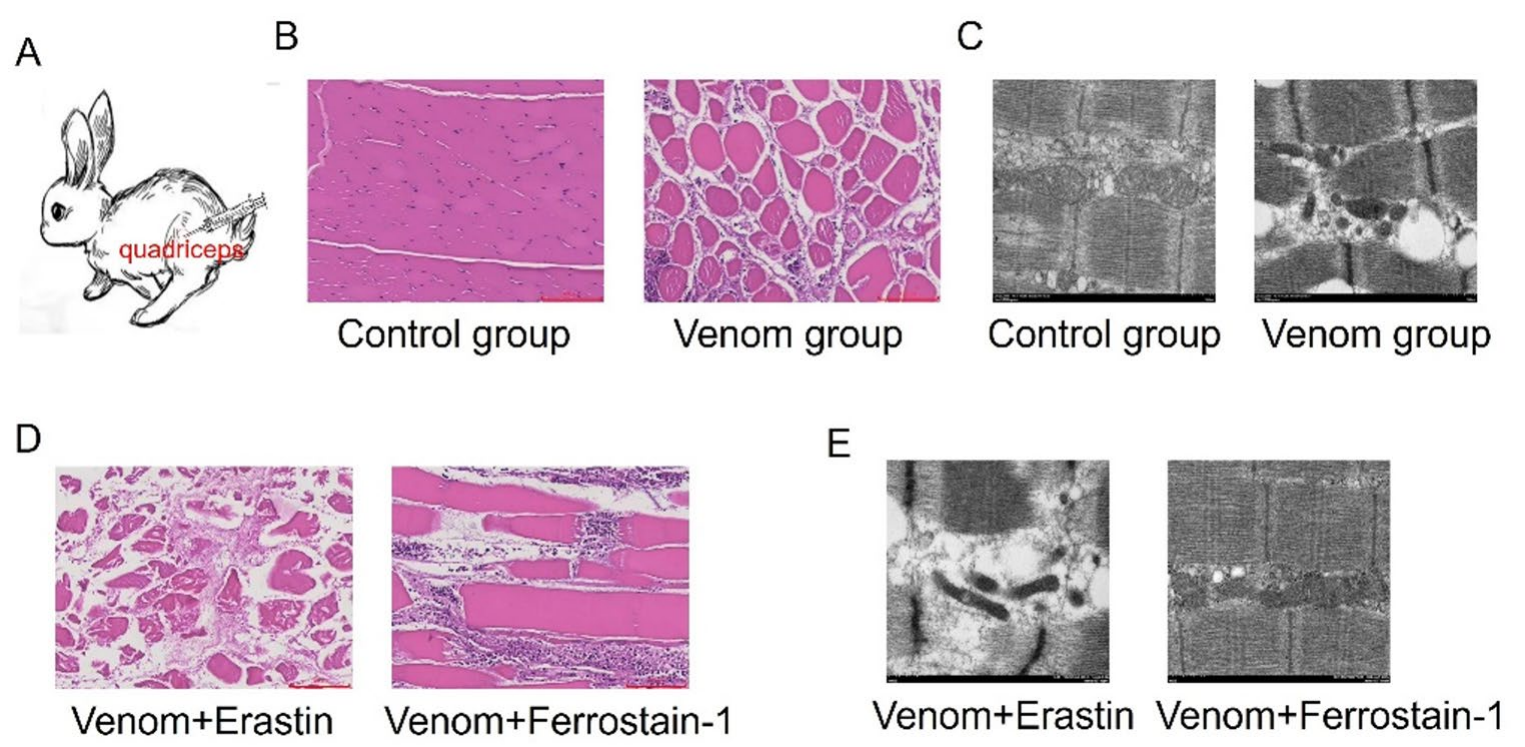
Establishment of rabbit model bitten by *T. stejnegeri.* **A** Schematic diagram of rabbit model bitten by *T. stejnegeri.* **B** HE staining showed morphological changes in skeletal muscle between control and venom group separately (magnification: 200×; scale bar = 50 μm). **C** Electron microscopy revealed ultrastructural alterations in skeletal muscle cells and mitochondria in control versus venom group (magnification: 10,000×; scale bar = 1 μm). **D** HE staining demonstrated skeletal muscle morphology changes in venom group treated with Erastin or Ferrostatin-1 separately (magnification: 200×; scale bar = 50 μm). **E** Electron microscopy analysis of skeletal muscle ultrastructure in venom group treated with Erastin or Ferrostatin-1(magnification: 10,000×; scale bar = 1 μm)

To establish a rabbit model of *T. stejnegeri* envenomation, we intramuscularly injected prepared 10 mg/mL *T. stejnegeri* venom solution into the right hindlimb of New Zealand white rabbits (Fig. 1A). HE staining and electron microscopy were conducted to validate successful model establishment. HE staining revealed that the control group exhibited normal skeletal muscle morphology with well-defined architecture, uniformly distributed nuclei, and regularly arranged muscle fibers, showing no evident necrosis or inflammatory cell infiltration. In marked contrast, the venom group displayed extensive myofibril dissolution and necrosis, severe structural disruption, inflammatory cell infiltration within the muscle interstitium; and nuclear condensation or disappearance (Fig. 1B). Electron microscopy analysis demonstrated that the control group maintained normal mitochondrial morphology with intact cristae structures and no observable swelling or membrane rupture. However, the venom group exhibited significant mitochondrial swelling, cristae fragmentation, and partial mitochondrial membrane disruption (Fig. 1C). These findings confirm the successful establishment of the *T. stejnegeri* venom-induced skeletal muscle injury model.

### Ferroptosis mediates *T. stejnegeri* venom-induced skeletal muscle injury

To elucidate the critical role of ferroptosis in *T. stejnegeri* venom-induced skeletal muscle injury, we treated venom group with either the ferroptosis inducer Erastin or inhibitor Ferrostatin-1. Histopathological analysis revealed that Erastin treatment exacerbated venom-induced muscle damage, demonstrating more extensive myofibril necrosis, severe fiber fragmentation, complete nuclear loss, and significantly increased inflammatory infiltration. These findings indicate that Erastin potentiates *T. stejnegeri* venom-induced skeletal muscle injury through ferroptosis activation. In contrast, Ferrostatin-1 treatment markedly attenuated muscle damage, showing better preserved fiber architecture and reduced inflammatory cell infiltration, demonstrating its protective effect through ferroptosis inhibition (Fig. 1D). Electron microscopy further confirmed these observations. The Erastin-treated group exhibited aggravated mitochondrial damage characterized by extensive membrane rupture and near-complete cristae disintegration. Conversely, Ferrostatin-1 treatment effectively mitigated mitochondrial damage, significantly reducing both swelling and cristae fragmentation (Fig. 1E). These results demonstrate that *T. stejnegeri* venom induces skeletal muscle necrosis primarily through ferroptosis activation. Importantly, ferroptosis inhibition by Ferrostatin-1 significantly ameliorates venom-induced damage, while ferroptosis induction by Erastin exacerbates the pathological changes.

### Molecular mechanisms of ferroptosis pathway in *T. stejnegeri* venom-induced skeletal muscle injury

**Fig. 2:**
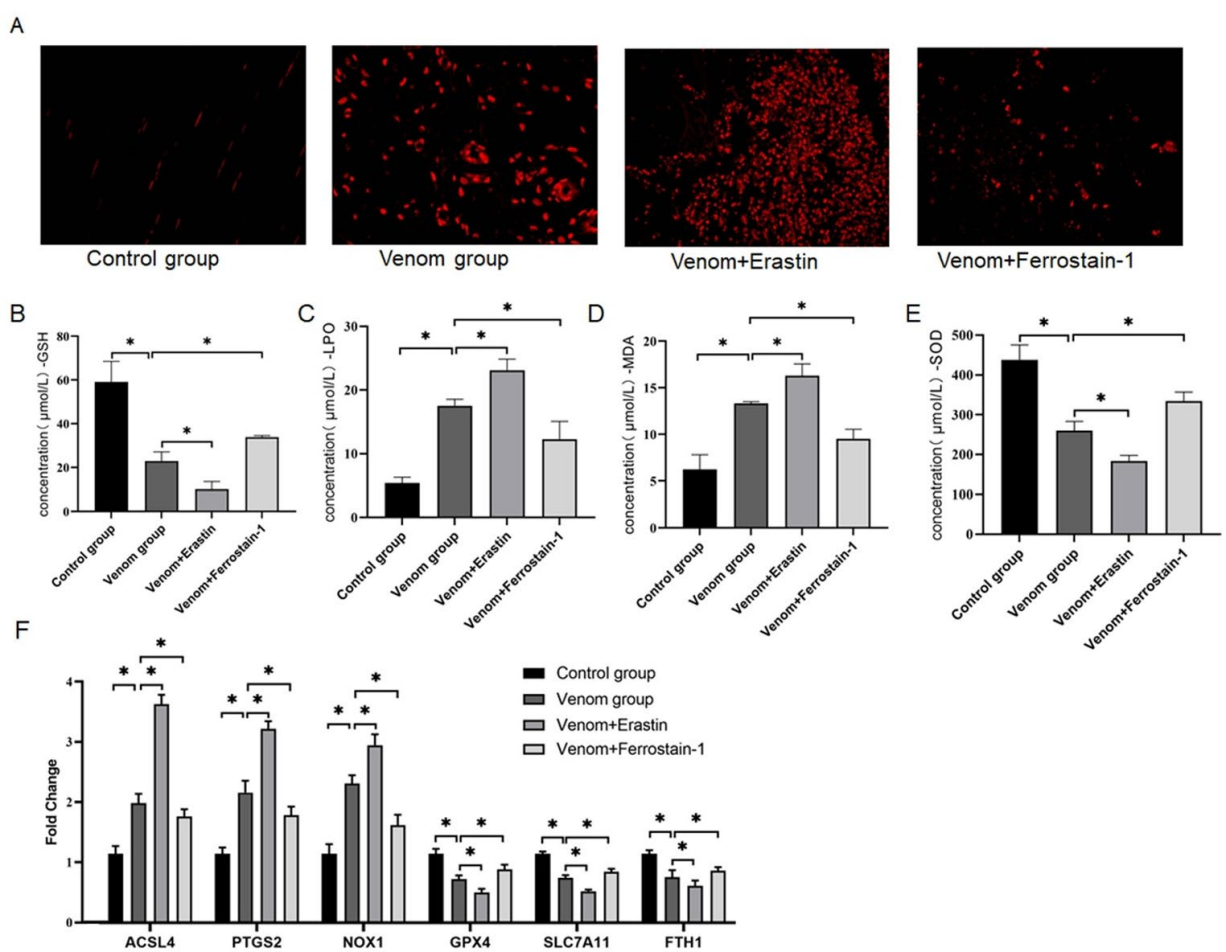
Ferroptosis mediates *T. stejnegeri* venom-induced skeletal muscle necrosis. **A**-**E** Determination of ROS, GSH, LPO, MDA and SOD levels in control, venom, venom+Erastin, and venom+Ferrostatin-1 groups separately. **F** qRT-PCR analysis of ferroptosis-related proteins (ACSL4, PTGS2, NOX1, GPX4, SLC7A11, FTH1) expression across experimental groups

To further elucidate the molecular mechanisms underlying ferroptosis in *T. stejnegeri* venom-induced skeletal muscle injury, we examined key indicators of the ferroptosis pathway. Quantitative analysis of reactive oxygen species (ROS) levels using DCFH-DA fluorescence staining revealed a significantly elevated intracellular ROS in the venom group compared to controls (*p* < 0.001). Notably, treatment with the ferroptosis inducer Erastin further exacerbated ROS accumulation, while the ferroptosis inhibitor Ferrostatin-1 markedly reduced ROS levels (Fig. 2A). Oxidative stress assessments showed characteristic ferroptosis alterations: GSH levels decreased by 70%, LPO levels increased fourfold, MDA levels increased threefold, and SOD levels decreased by 50% (Fig. 2B-E). Importantly, ferroptosis modulators produced distinct effects - Ferrostatin-1 treatment mitigated, whereas Erastin exacerbated, these oxidative stress markers, biochemically confirming the central role of ferroptosis in *T. stejnegeri* venom-induced injury. At the molecular level, qRT-PCR analysis showed that the venom group exhibited approximately 2-fold upregulation of pro-ferroptosis genes (ACSL4, PTGS2, NOX1) and 50% downregulation of anti-ferroptosis genes (GPX4, SLC7A11, FTH1). Ferrostatin-1 treatment effectively reversed these expression changes, while Erastin produced opposing effects (Fig. 2F). These findings further demonstrate that T. stejnegeri venom induces skeletal muscle injury by dysregulating ferroptosis-related gene expression and elevating oxidative stress levels.

### Protective effects of snakebite capsules against *T. stejnegeri* venom-induced injury

**Fig. 3:**
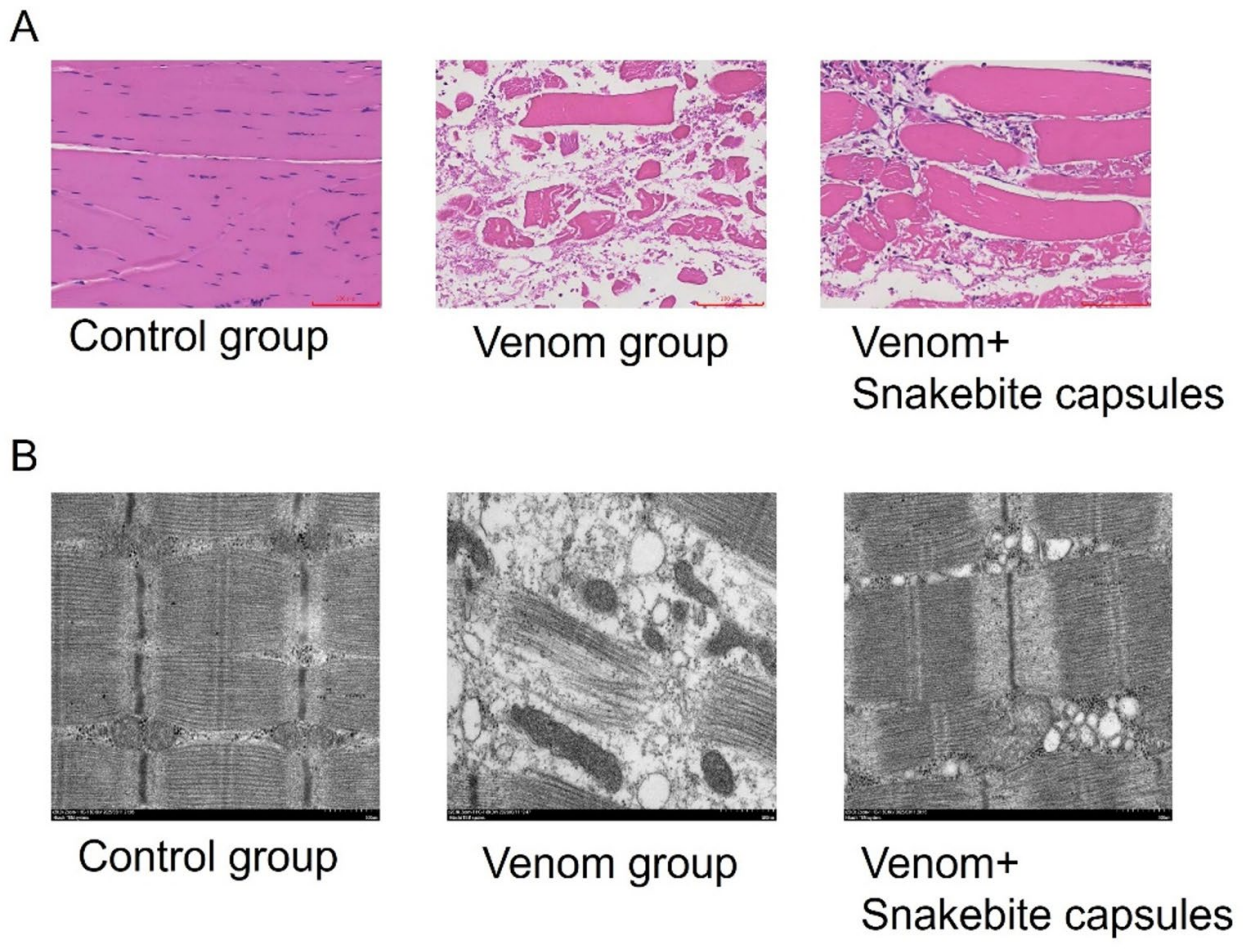
Therapeutic effects of Snakebite capsules on skeletal muscle necrosis. **A** HE staining comparing skeletal muscle morphology in the control, venom, and venom+Snakebite capsules groups separately (magnification: 200×; scale bar = 50 μm). Data are presented as mean ± SD (*n* = 3). ***p < 0.001 vs. control (one-way ANOVA/Tukey’s test). **B** Electron microscopy evaluation of skeletal muscle cells and mitochondria in the control, venom, and venom+Snakebite capsules groups (magnification: 200×; scale bar = 50 μm)

Having established the role of ferroptosis in *T. stejnegeri* venom-induced skeletal muscle injury, we further investigated whether Snakebite Capsules could exert therapeutic effects by targeting the ferroptosis pathway. Histopathological examination was performed on envenomed rabbits treated with Snakebite Capsules solution. HE staining demonstrated that Snakebite Capsules treatment significantly attenuated skeletal muscle necrosis (qualitative observation): compared to the venom group, the muscle fiber alignment was better preserved, the degree of myofibril dissolution was reduced, and the number of inflammatory cells infiltrating the muscle interstitium was decreased. (Fig. 3A), indicating the Snakebite capsule’s protective effect against *T. stejnegeri* venom-induced skeletal muscle damage. Ultrastructural analysis by electron microscopy revealed that Snakebite Capsules treatment effectively maintained mitochondrial integrity, showing markedly reduced swelling and cristae disruption (Fig. 3B). These findings demonstrate that Snakebite Capsules can effectively ameliorate *T. stejnegeri* venom-induced skeletal muscle injury and preserve mitochondrial structure. Notably, the therapeutic efficacy of Snakebite Capsules showed a similar trend of improvement to that of the positive control Ferrostatin-1. Statistical analysis showed that both treatments significantly reduced muscle fiber necrosis compared to the venom group (*p* < 0.001), and there was no significant difference in the reduction rate of key ferroptosis indicators (e.g., MDA, LPO) between the two groups (*p* > 0.05), suggesting that the protective mechanism of Snakebite Capsules may involve inhibition of the ferroptosis pathway.

### Snakebite capsules exert a protective effect by inhibiting the ferroptosis pathway

**Fig. 4:**
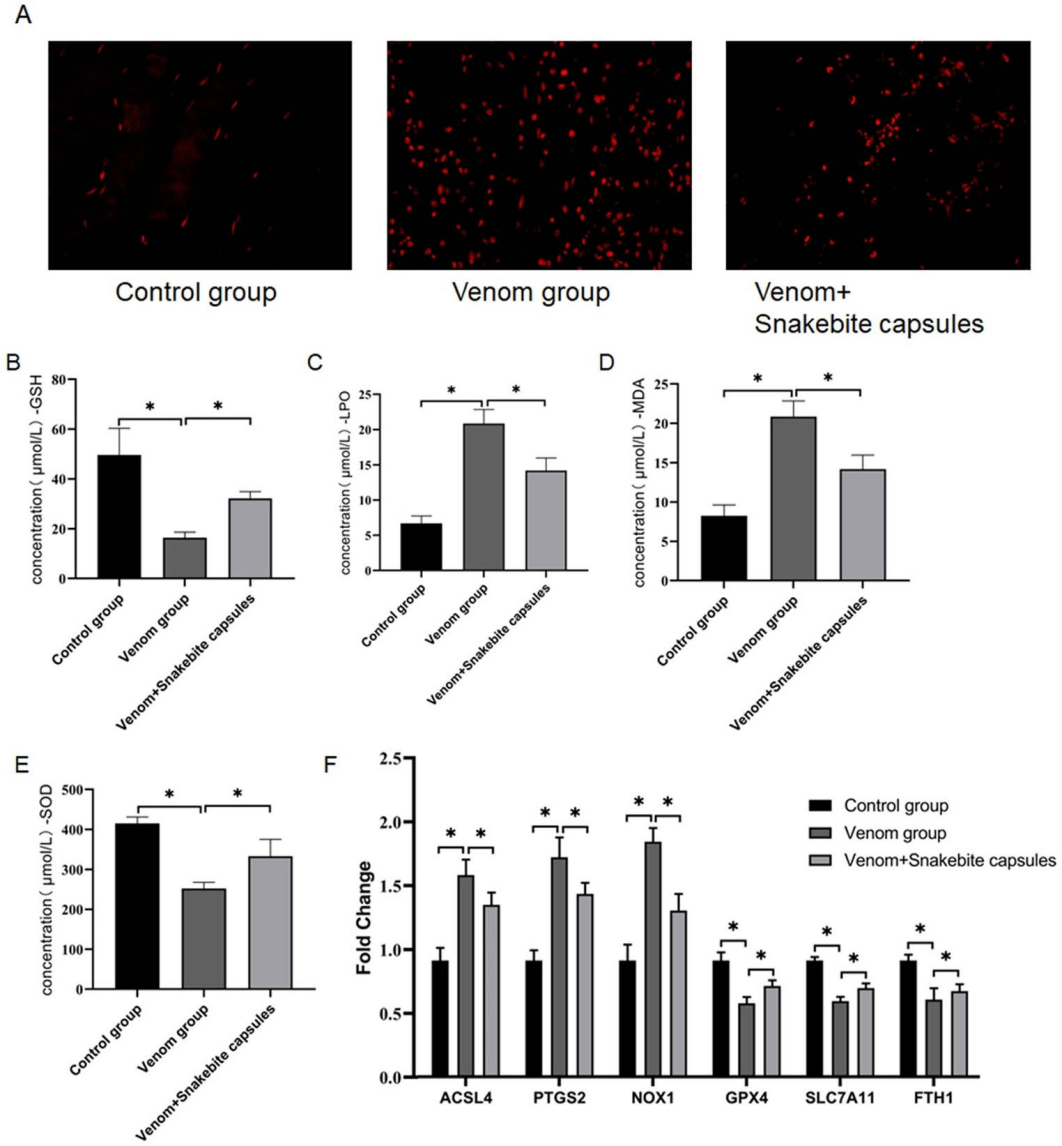
Snakebite capsules ameliorate skeletal muscle necrosis by inhibiting ferroptosis pathway. **A**-**E** Measurement of ROS, GSH, LPO, MDA and SOD levels in control, venom, and venom+Snakebite capsules groups separately. **F** qRT-PCR analysis of ferroptosis-related proteins (ACSL4, PTGS2, NOX1, GPX4, SLC7A11, FTH1) expression patterns

To elucidate the therapeutic mechanism of Snakebite Capsules in greater depth, we systematically examined its effects on ferroptosis-related biomarkers. Snakebite Capsules reduced ROS by ~ 50%, restored GSH to 60%, suppressed LPO/MDA by 25%/30%, and restored SOD to 80% of control (all *p* < 0.05) (Fig. 4A-E). At the molecular level, Snakebite Capsules treatment markedly downregulated pro-ferroptosis genes (ACSL4, PTGS2, and NOX1), while upregulating anti-ferroptosis genes (GPX4, SLC7A11 and FTH1) (Fig. 4F). This bidirectional regulatory effect provides compelling evidence for its protective mechanism through modulation of the ferroptosis pathway.

**Fig. 5:**
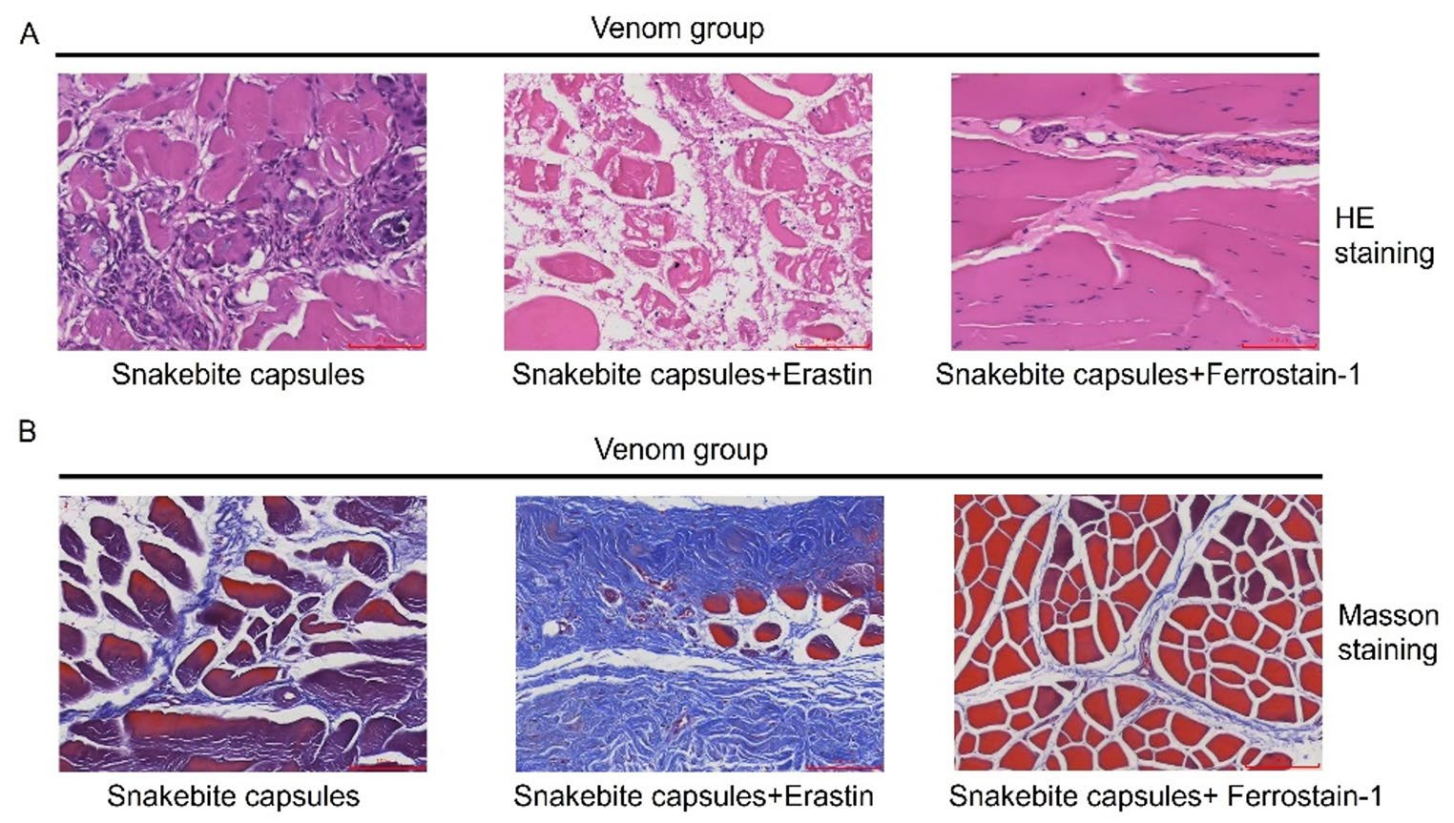
Rescue experiments demonstrating the regulatory relationship between Snakebite capsules and ferroptosis. **A** HE staining of skeletal muscle morphology in Snakebite capsules, Snakebite capsules+Erastin, and Snakebite capsules+Ferrostatin-1 groups separately (magnification: 200×; scale bar = 50 μm). **B** Masson staining showing collagen fiber organization in Snakebite capsules, Snakebite capsules+Erastin, and Snakebite capsules+Ferrostatin-1 groups (magnification: 200×; scale bar = 100 μm)

To further validate the protective effects of Snakebite Capsules through modulation of the ferroptosis pathway, we conducted rescue experiments. HE staining revealed that the Snakebite Capsules-treated group exhibited well-preserved skeletal muscle morphology with properly aligned muscle fibers and significantly reduced inflammatory cell infiltration, however, co-administration with the ferroptosis inducer Erastin completely abolished the protective effects of Snakebite Capsules, resulting in exacerbated myofiber fragmentation and increased inflammatory infiltration. In contrast, when combined with the ferroptosis inhibitor Ferrostatin-1, the therapeutic efficacy of Snakebite Capsules was markedly enhanced, with nearly complete prevention of myofiber necrosis and further reduction of inflammatory responses (Fig. 5A). Masson staining demonstrated that Snakebite Capsules treatment alone maintained relatively organized muscle fiber arrangement with mild fibrosis. Co-treatment with Erastin showed severe muscle fiber disruption, significantly increased fibrosis, and extensive collagen deposition. Conversely, combination with Ferrostatin-1 resulted in better preserved muscle fiber alignment and further attenuated fibrosis (Fig. 5B). These findings not only confirm that Snakebite Capsules exert their protective effects through inhibition of the ferroptosis pathway, but also elucidate a synergistic interaction with ferroptosis inhibitors and an antagonistic relationship with ferroptosis inducers. Erastin’s exacerbation of injury was specifically reversed by Ferrostatin-1, a ferroptosis-specific inhibitor. This confirms its pro-injury effect is primarily mediated by ferroptosis activation, not off-target effects (Sun et al. 2022). The results provide crucial mechanistic insights and inform potential therapeutic strategies for clinical applications.

## Discussion

*Trimeresurus stejnegeri* venom, primarily hematotoxic, induces pathological processes including coagulation disorders, hyperfibrinolysis, and tissue damage (Wang et al. 2025; Qiu et al. 2024; Chen et al. 2025). Traditional treatment methods heavily rely on anti-snake venom serum(Chuang et al. 2024), which neutralizes toxic components but has limitations (insufficient specificity, side effects)(Hung et al. 2024), while also moderately reducing the level of uric acid (Xie et al. 2023). However, the use of anti-snake venom serum is associated with certain limitations, such as insufficient specificity, prominent side effects, and inadequate supply in some regions (Sasidharan et al. 2025). Therefore, finding safer and more effective alternative or adjunctive treatment options is of significant importance. Snakebite capsules, a traditional Chinese medicine formulation, demonstrates significant clinical efficacy in *T. stejnegeri* bites. Its therapeutic benefits likely stem from improving platelet activation and protecting vascular endothelial cells, while also exhibiting anti-inflammatory, antioxidant, and tissue-repair properties that confer unique therapeutic advantages.

Emerging evidence implicates ferroptosis as a potential therapeutic target in various diseases. Ferroptosis and inflammation are mutually reinforcing: ferroptosis induces inflammatory responses, which in turn promote ferroptosis. Our observed inflammatory infiltration is likely secondary to ferroptosis, and Snakebite Capsules may indirectly alleviate it via ferroptosis inhibition(Sun et al. 2024; Zeitler et al. 2021). Given that snake venoms contain multiple toxins (e.g., PLA2s, metalloproteinases, hemolysins) capable of inducing ferroptosis, and drawing analogies from other animal toxins, we hypothesized its potential involvement in *T. stejnegeri* envenomation. Our study confirmed ferroptosis via multi-dimensional evidence: (1) characteristic biomarker changes, (2) ferroptosis-related gene dysregulation, (3) rescue by ferroptosis modulators. We found that T. stejnegeri envenomation triggered hallmark features of ferroptosis in skeletal muscle, including significantly elevated ROS, decreased GSH, increased MDA/LPO, and reduced SOD activity. Furthermore, qRT-PCR analysis revealed upregulated pro-ferroptotic genes (ACSL4, PTGS2, NOX1) alongside downregulated anti-ferroptotic genes (SLC7A11, GPX4, FTH1), confirming that the venom induces skeletal muscle damage by activating the ferroptosis pathway.

Against this established mechanism, our study elucidated the Snakebite capsule’s mechanism through ferroptosis inhibition. Ferroptosis, an iron-dependent programmed cell death characterized by uncontrolled lipid peroxidation, involves GSH depletion and GPX4 inactivation (Ursini and Maiorino 2020), leading to lethal accumulation of lipid peroxides that disrupt cellular membranes (Yang et al. 2023). Our findings demonstrated that Snakebite Capsules significantly reduce ROS generation, elevate GSH levels, decreases MDA and LPO content, and enhances SOD activity, thereby mitigating venom-induced oxidative damage. Furthermore, it modulates ferroptosis-related genes, downregulating ACSL4, PTGS2 and NOX1 (Ali et al. 2024) while upregulating SLC7A11, GPX4 and FTH1 expression. These results substantiate the capsule’s protective role in part through ferroptosis pathway inhibition.

We acknowledge the 24 h post-envenomation administration is later than clinical practice (6–12 h). This targeted ferroptosis peak phase, but may limit clinical generalizability. Future studies will compare multiple administration time windows to clarify optimal intervention timing. However, Snakebite Capsules are a clinically approved traditional Chinese medicine formulation with a well-documented safety profile. Previous clinical studies have shown that this formulation does not cause obvious adverse reactions in normal tissues (Kumar et al. 2025; Gupta et al. 2024). The therapeutic dose used in this study (0.348 g/kg) is consistent with the clinical recommended dose, and there is no evidence that this dose would alter the oxidative stress or ferroptosis status of normal skeletal muscle.

In this study, we used serum GSH, MDA, and LPO levels as indicators to evaluate skeletal muscle injury. This is based on the fact that ferroptosis induced by skeletal muscle injury will lead to the release of oxidative stress products and antioxidant molecules into the bloodstream, and previous studies have confirmed that the serum levels of these biomarkers are significantly correlated with the degree of local muscle tissue damage(Sun et al. 2023). In addition, the changes in serum biomarkers in this study are consistent with the morphological observations of skeletal muscle (e.g., myofibril dissolution, inflammatory infiltration), which further verifies the representativeness of serum biomarkers for local muscle pathology. Nano-encapsulation may enhance Snakebite Capsules’ bioavailability(Majumdar et al. 2024). The synergistic effect with Ferrostatin-1 highlights potential for combination therapy in clinical practice.

In summary, Snakebite capsule’s multi-target mechanism offers distinct therapeutic advantages-ameliorating coagulation disorders while reducing oxidative stress and tissue damage through ferroptosis inhibition, presenting novel approaches for *T. stejnegeri* envenomation management(Mayfield et al. 2025). The integration of biotechnological approaches in formulation optimization and delivery systems could significantly enhance the clinical translation and efficacy of this promising therapeutic strategy (Julve Parreño et al. 2018).

## Conclusion

**Fig. 6:**
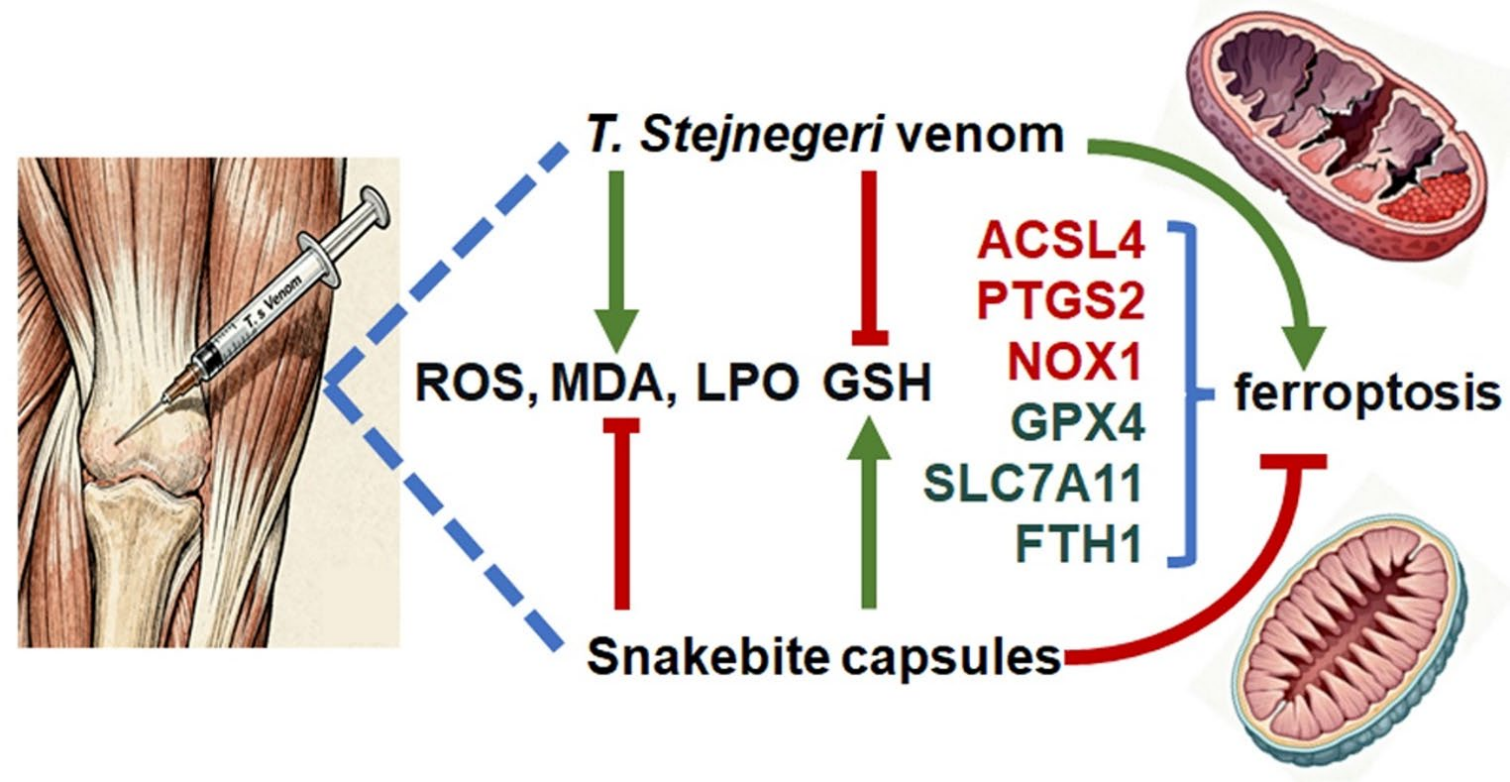
Proposed mechanism of T. stejnegeri venom-induced skeletal muscle ferroptosis and the therapeutic action of Snakebite Capsules. The venom triggers ferroptosis, characterized by mitochondrial damage, glutathione (GSH) depletion, and elevated oxidative stress (ROS/LPO/MDA), leading to myofiber necrosis. Snakebite Capsules confer protection by upregulating key anti-ferroptosis defenses (GPX4, SLC7A11, GSH) and suppressing pro-ferroptosis factors (ACSL4, PTGS2, NOX1), thereby inhibiting lipid peroxidation, preserving mitochondrial integrity, and ultimately attenuating skeletal muscle injury

In conclusion, our findings demonstrate that ferroptosis plays a critical role in the pathogenesis of *T. stejnegeri* venom-induced skeletal muscle injury, characterized by oxidative stress, lipid peroxidation, and mitochondrial dysfunction. Snakebite Capsules effectively ameliorate tissue damage at least partially through modulation of the ferroptosis pathway, as evidenced by restored redox homeostasis and regulation of key ferroptosis-related genes. These results provide a mechanistic basis for the clinical application of Snakebite Capsules and highlight ferroptosis as a promising therapeutic target for snakebite envenomation (Fig. 6).

### Study limitations

Although this study demonstrates the involvement of ferroptosis in *T. stejnegeri* envenomation and the therapeutic potential of Snakebite capsules, several limitations should be acknowledged.

First, the sample size was relatively small (with only 3 animals per group) due to limitations in experimental resources and animal ethics. A power analysis via G*Power 3.1 software validated this size, with 3 animals per group achieving 80% statistical power (α = 0.05) for detecting intergroup differences in key ferroptosis indicators (MDA, GSH) and meeting basic requirements for this animal study type. However, the small sample may reduce the statistical stability and generalizability of partial results, and future studies will expand the sample size to enhance finding robustness.

Second, the assessment of Hematoxylin-Eosin and Masson staining was limited to qualitative observation to identify trends. Although this approach provides directional insights, the lack of quantitative data precludes a more in-depth analysis. We maximized observational objectivity by detailing morphological tissue changes, and future studies will adopt standardized quantitative staining analysis to quantify tissue damage and therapeutic effects accurately.

Third, this study assessed the expression of ferroptosis-related genes using qRT-PCR, while it lacks further validation at the protein level. This limitation arises from the limited availability of rabbit-specific antibodies and the initial research focus on core ferroptosis pathway verification. Future studies will optimize antibody screening to supplement protein-level detection and further elucidate the underlying molecular regulatory mechanisms.

Fourth, Snakebite Capsules were administered at 24 h post-envenomation, later than the typical 6–12 h clinical intervention window. This timing was designed to target the 24–48 h peak of ferroptosis post-envenomation, yet the discrepancy with clinical practice may limit the direct translational generalizability of the results. Future research will set up multiple administration time groups to compare therapeutic effects and define the optimal clinical intervention window for the capsules.

## Data Availability

The raw data supporting the conclusions of this article will be made available by the authors, without undue reservation, to any qualified researcher.

## Abbreviations

*T. stejnegeri*: *Trimeresurus stejnegeri*
HE staining: Hematoxylin and eosin staining
ROS: Reactive oxygen species
GSH: Glutathione
LPO: Lipid peroxide
MDA: Malondialdehyde
SOD: Superoxide dismutase
qRT-PCR: Quantitative real-time polymerase chain reaction
PTGS2: Prostaglandin-endoperoxide synthase 2
ACSL4: Acyl-CoA synthetase long chain family member 4
NOX1: NADPH oxidase 1
GPX4: Glutathione peroxidase 4
SLC7A11: Solute carrier family 7 member 11
FTH1: Ferritin heavy chain 1
